# The Role of Bacteria in KSHV Infection and KSHV-Induced Cancers

**DOI:** 10.3390/cancers13174269

**Published:** 2021-08-25

**Authors:** Ashley Markazi, Wen Meng, Paige M. Bracci, Michael S. McGrath, Shou-Jiang Gao

**Affiliations:** 1 Cancer Virology Program, UPMC Hillman Cancer Center, Pittsburgh, PA 15213, USA; admarkazi@gmail.com (A.M.); mengw@upmc.edu (W.M.); 2 Department of Microbiology and Molecular Genetics, University of Pittsburgh School of Medicine, Pittsburgh, PA 15219, USA; 3 Department of Epidemiology and Biostatistics, University of California at San Francisco, San Francisco, CA 94158, USA; paige.bracci@ucsf.edu; 4 Department of Laboratory Medicine, Pathology and Medicine, University of California at San Francisco, San Francisco, CA 94143, USA; Mike.McGrath@ucsf.edu

**Keywords:** Kaposi’s sarcoma, KSHV, microbiome, HIV, opportunistic infection

## Abstract

**Simple Summary:**

The aim of this article is to review the complex interactions of bacteria with Kaposi’s sarcoma-associated herpesvirus (KSHV) infection and KSHV-induced cancers. KSHV is causally associated with multiple cancers including Kaposi’s sarcoma (KS) and primary effusion lymphoma. Among patients coinfected by HIV and KSHV, patients with KS have a distinct oral microbiome compared to patients without KS. Moreover, KSHV patients have increased levels of salivary bacterial pathogen-associated molecular patterns compared to KSHV-negative patients. KSHV-associated bacterial species can increase KSHV replication and dissemination, and enhance cell proliferation of KSHV-transformed cells. The analysis of bacterial biomarkers associated with KSHV may help improve our understanding of the mechanisms driving KSHV-induced oncogenesis and identify novel targets for improving therapies of KSHV-related cancers.

**Abstract:**

The objective of this article is to review the current status of the bacteria-virus interplay in Kaposi’s sarcoma-associated herpesvirus (KSHV) infection and KSHV-driven cancers. KSHV is the etiological agent of several cancers, including Kaposi’s sarcoma (KS) and primary effusion lymphoma. Due to immunosuppression, patients with KSHV are at an increased risk for bacterial infections. Moreover, among patients coinfected by HIV and KSHV, patients with KS have distinct oral microbiota compared to non-KS patients. Bacterial biomarkers associated with KSHV-driven cancers can provide insights in discerning the mechanisms of KSHV-induced oncogenesis. For example, pathogen-associated molecular patterns and bacterial products of certain bacterial species can regulate the expression of KSHV lytic and latent genes, thereby affecting viral replication and dissemination. In addition, infection with distinct opportunistic bacterial species have been associated with increased cell proliferation and tumorigenesis in KSHV-induced cancers through activation of pro-survival and -mitogenic cell signaling pathways. By elucidating the various mechanisms in which bacteria affect KSHV-associated pathogenesis, we will be able to pinpoint therapeutic targets for KSHV infection and KSHV-related cancers.

## 1. Introduction

The utilization of bacterial markers to determine cancer pathology has progressed tremendously in recent years. Advances in technology have empowered researchers to more thoroughly elucidate complex interactions between the microbiome and cancer. Microbiome analyses using next generation sequencing report that the colonization of pathogenic bacterial species is increased in cancer patients and exacerbates cancer pathogenesis [1]. Pathogenic bacteria such as *Fusobacterium nucleatum* and *Helicobacter pylori* have been extensively studied for their carcinogenic effects, as they can produce toxins and effector proteins that induce host cell damage and alter cell signaling pathways involved in cell survival and proliferation [2]. Contrarily, many studies have shown that certain bacteria such as probiotics have anti-inflammatory effects that may aid in cancer prevention [3]. Moreover, recent studies have shown that bacteria can interact with cancer-causing viruses to promote viral replication and the progression of viral cancers [4]. The analysis of bacteria-virus interactions is paramount to microbiome-cancer studies, as up to 15% of cancers have a viral etiology [5]. This review will specifically focus on the effects of bacteria on infection of Kaposi’s sarcoma-associated herpesvirus (KSHV) and its associated cancers, which develop most frequently in immunosuppressed individuals. To thoroughly comprehend the interactions between bacteria and KSHV-induced cancers, it is necessary to begin with a brief overview of the general interactions between the microbiome, immune response, and cancer.

## 2. Bacterial Infection and the Innate Immune Response: Effects on Cancer Pathology

The human immune system responds to bacterial infection by producing inflammation. Bacteria possess pathogen-associated molecular patterns (PAMPs) that are recognized by pattern recognition receptors (PRRs) expressed by both non-immune and immune cells [6,7]. Activation of PRRs causes a downstream cascade of signal transduction pathways, resulting in enhanced expression of inflammatory cytokines [6]. Inflammatory cytokines amplify inflammation by signaling additional immune cells to migrate towards the infection [6]. Immune cells, in addition to producing more cytokines, respond to infection by generating antibacterial substances including reactive oxygen species (ROS) or reactive nitrogen species (RNS), which can cause apoptosis in infected cells [8]. Bacterial infection can also activate the complement system, resulting in induction of inflammatory mediators, increased angiogenesis, and migration of immune cells to the infection [9]. Although inflammation is necessary for controlling infection, unregulated or continuous inflammation can initiate and/or increase carcinogenesis [10].

A number of studies have annotated the pathways linking PRR activation and cancer cell proliferation [11]. Bacterial PAMPs can activate transmembrane and cytosolic PRRs [6]. Transmembrane receptors include C-type lectins receptors and toll-like receptors (TLRs) that recognize extracellular and endosomal-derived PAMPs [12,13]. Cytosolic receptors include nucleotide-binding domain leucine-rich repeat containing receptors (NLRs) and RIG-I like receptors (RLRs) that recognize intracellular infections and damage-associated molecular patterns (DAMPs) [6]. Activation of these PRRs causes induction of inflammatory pathways including the NF-κB pathway and mitogen activated protein kinase (MAPK) pathways which increase expression of cytokines such as IL-10, TNF-α, IL-1β, and IL-6 [6]. The oldest of PRRs are the TLRs, which recognize bacterial structure components. For examples, lipopolysaccharides (LPS) are detected by TLR4, lipoproteins are detected by TLR1/2 or TLR2/6 heterodimers, and flagellin is detected by TLR5 [6]. Studies have demonstrated that bacterial ligands including LPS, peptidoglycans, and flagellin promote cell proliferation and/or invasiveness in several cancer cell lines [14,15,16,17,18]. Moreover, some studies suggest that increased levels of circulating bacterial PAMPs are correlated with a higher cancer risk [19,20,21].

However, contradictory studies show decreased cell proliferation in cells stimulated with bacterial PAMPs [22,23,24]. Moreover, bacteria and their PAMPS have been studied as potential therapies for decreasing tumors [25,26]. These contradictory effects are likely due to the multiple responses of cells to TLR activation or differences within the tumor environment (such as compositions of immune cells) [22,27,28,29]. Elucidating the factors that direct the downstream signaling of TLRs toward pro-survival versus apoptotic pathways will be especially important for determining how to best utilize bacteria for cancer therapy.

Due to the substantial evidence of bacterial impact on cancer pathology, it is essential to delineate the intricate interactions of the bacterial microbiome with cancer. The microbiome consists of the microbial community, which maintains intricate interactions. Their effects on the habitat are complex [30]. The human body is home to a multitude of different types of microbiomes, each with a distinct composition of microbial species [31]. The intestines and oral cavities harbor the greatest number and diversity of species [31]. Cancerous tumors and lesions also have distinct microbiomes, consisting of intracellular bacterial species highly specific to the particular tumor type [32]. The species’ composition and bacterial interactions dictate the types of bacterial metabolites, biofilm, and quorum signaling molecules produced within the microbiome [30]. In addition to being parts of the bacterial structures, bacterial products have a significant effect on immune response [33]. The bacterial microbiome can vary greatly based on many genetic and environmental factors, including the host’s diet, location, weight, and ethnicity [34]. Based on the current literature, microbial effects on cancer can be broadly classified into three major categories: (1) dysregulation of host immunity induced by bacterial PAMPs, (2) direct interactions between bacterial products and cancer cells, (3) indirect mechanisms involving bacterial products, and (4) for viral cancers, direct interactions between bacteria and bacterial products, and viruses.

## 3. KSHV and KSHV-Associated Cancers

This review focuses on the effects of bacteria on KSHV infection and KSHV-associated cancers. KSHV is the etiological agent of Kaposi’s sarcoma (KS) and primary effusion lymphoma (PEL) [35]. Although KSHV infection has higher prevalence in the homosexual population, infection rates vary in the general population according to geographic regions [36]. Its infection rate is the highest in some African regions (reaching 70%), median in Eastern European and Mediterranean regions (in the range of 20–30%), and lowest in North America and most other European and Asian countries (in the range of 4–12%) [37]. Importantly, most individuals with KSHV infection do not develop any KSHV-associated cancers, implying that additional co-factors are necessary for KSHV-infected subjects to develop cancer [38].

As a gammaherpesvirus, KSHV causes life-long persistent infection [39]. The KSHV latency phase is characterized by the expression of limited viral latent genes that helps the virus escape from the host’s immune detection [40]. The lytic phase consists of highly orchestrated expression of viral lytic genes that lead to viral DNA replication and virion release [40]. Lytic replication is essential for both transmission and dissemination of the virus [40]. The majority of viral genes are lytic and are silenced during latency through multiple epigenetic modifications of the viral genome including DNA methylation, and histone deacetylation and repressive histone methylation [40]. Physiological factors that activate KSHV lytic replication include immunosuppression, hypoxia, inflammation, and co-pathogenic infections [4].

Since not all individuals infected with KSHV develop malignancies, it is especially important to understand the roles co-factors play in the development and severity of KSHV-induced cancers. KSHV most commonly causes cancers in immunocompromised individuals, especially those with HIV infection [35]. Patients with HIV have decreased CD4+ T cell counts, which plays a crucial role in controlling KSHV lytic replication and KS tumors [41,42]. HIV proteins TAT (trans-activator of transcription), Nef, and Vpr can directly interact with and regulate the functions of KSHV proteins in addition to altering host immune regulation, leading to promotion of KSHV infection [43,44,45,46,47,48]. Studies have shown that low CD4+ T cell count may increase the risk of developing KS and PEL, further indicating the significance of immunosuppression on KSHV-induced pathologies [4].

**Figure 1:**
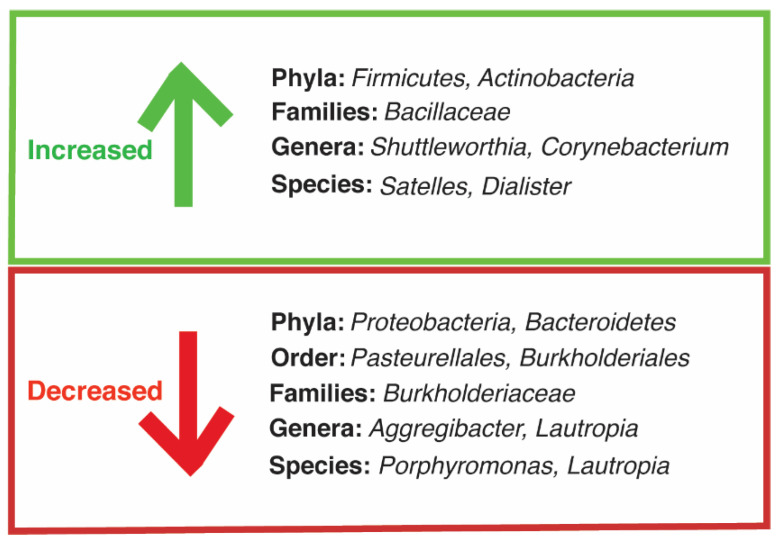
Observed increases and decreases in phyla, orders, families, genera, and species of oral microbiota in HIV/KSHV-coinfected oral KS patients compared with HIV/KSHV-coinfected patients without oral KS or oral KSHV. Figure was modified from Figure 5 in the open access article by Gruffaz et al. [51].

Suppressed immunity associated with HIV increases the risk of opportunistic infections [4]. Several studies show that microbiomes of immunosuppressed patients have a lower overall diversity of bacterial species and increased colonization of pathogenic species compared to healthy individuals [49,50]. Oral bacterial infection, in particular, is highly relevant in KS patients, as periodontal disease is present in higher frequency in KS patients than healthy individuals [4]. Gruffaz et al. used next generation sequencing to analyze the microbiota in saliva samples of KS patients with either oral KS, no oral KS but oral cell-associated KSHV, or neither oral KS nor oral cell-associated KSHV DNA [51]. Oral KS patients had the most phylogenetically distant composition of bacteria compared with the two other groups [51]. Figure 1 depicts the observed increases and decreases of oral microbiota in the oral KS group compared with the HIV/KSHV-coinfected patients without oral KS or oral KSHV [51]. Oral KS patients had higher levels of the phyla *Firmicutes* and *Actinobacteria* and decreased levels of the phyla *Bacteroidetes* and *Proteobacterium*. Moreover, oral KS patient samples had higher levels of common oral pathogens including bacteria in the genera *Corynebacterium* and *Shuttleworthia* [51]. These results strongly imply that oral microbiota interaction with HIV/KSHV coinfection may play an important role in influencing the development of oral KS. Other studies have also detected a positive correlation between herpesvirus and periodontal pathogens, including *Porphyromonas. gingivalis* and *Fusobacterium nucleatum* [52]. Moreover, many studies have reported that common oral pathogens produce metabolites that reactivate KSHV and Epstein–Barr virus (EBV), another oncogenic gammaherpesvirus, resulting in enhanced viral infectivity and promotion of lytic replication of the virus [53,54,55].

## 4. Impact of Bacteria on KSHV Lytic Replication

Butyrate, a common metabolic end product of certain bacterial species, has been shown to reactivate herpesviruses including KSHV [55]. Butyrate inhibits the activities of histone deacetylases (HDACs) and promotes hyperacetylation, leading to increased viral gene expression [56]. Multiple studies have shown that the medium of butyrate-producing oral pathogens can result in KSHV reactivation [53,55]. A study by Morris et al. explored the role of microbial infection in KSHV reactivation by treating PEL cell line BCBL1 cells with spent medium from oral disease pathogens including *P. gingivalis*, *F. nucleatum*, *Prevotella intermedia*, and *Streptococcus mutans* [55]. Authors detected increased viral lytic gene expression in cells treated with mediums from the potent butyrate-producing bacteria, namely the *P. gingivalis* and *F. nucleatum* medium. The study also examined the mechanism of bacterial-induced reactivation and reported that activation of viral lytic genes by the bacterial mediums occurred through the p38 MAPK pathway as this pathway has been shown to be both essential and sufficient for KSHV lytic replication during primary infection and reactivation [55,57,58,59,60].

Yu et al. further explored the effects of medium from *F. nucleatum* and *P. gingivalis* on KSHV reactivation [53]. Their study reported increased expression of viral lytic gene expression in BCBL1 cells treated with medium from *P. gingivalis* and *F. nucleatum*. Moreover, treatment of BCBL1 cells with different doses of pure individual short chain fatty acids (SCFAs) detected in the mediums of *P. gingivalis* and *F. nucleatum* except acetic acid resulted in increased expression of lytic gene RTA (ORF50) [53]. Treatment with butyric acid resulted in the greatest effect, whereas an additive effect was observed when SCFAs were combined [53]. Interestingly, by analyzing levels of SCFAs in saliva, Yu and colleagues found higher concentrations of salivary SCFAs from *P. gingivalis* and *F. nucleatum* in patients with periodontal diseases compared to healthy controls [53]. Yu et al. explored the mechanism of KSHV reactivation by bacteria, reporting that BCBL1 cells, KSHV-infected human normal epithelial cells (HOECs), and KSHV-infected human telomerase-immortalized human umbilical vein endothelial cells (TIVE-KSHV) treated with *P. gingivalis* and *F. nucleatum* had significantly reduced expression of class 1/2 HDACs. In contrast, no effect was seen with *Escherichia coli* medium [53]. These findings provide evidence that SCFAs from periodontal pathogens might reactivate KSHV in the oral cavity and therefore influence the development and progression of KSHV-induced cancers.

**Figure 2:**
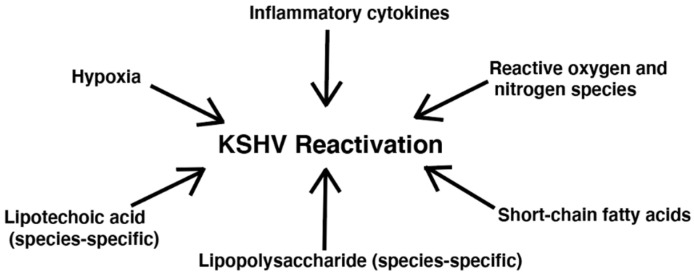
Bacteria-related factors that reactivate KSHV.

Besides SCFA production, additional bacterial effects have been linked to KSHV reactivation, including inflammation related to cytokines and ROS [42]. Figure 2 depicts bacteria-related factors that have been shown to reactivate KSHV. Ye et al. observed that both exogeneous and endogenous hydrogen peroxide (H_2_O_2_) increased KSHV lytic gene expression in HUVEC and PEL cell lines [61]. Although, at higher concentrations, H_2_O_2_ causes apoptosis and senescence in primary cells, at lower concentrations, H_2_O_2_ can activate multiple redox signaling pathways such as the MAPK pathways [61]. Ye et al. reported that H_2_O_2_ induction of KSHV reactivation depended on ERK1/2, JNK, and p38 pathways [61]. As H_2_O_2_ can be produced directly by bacteria and indirectly by bacterial stimulation of immune cells, it may be of interest to more thoroughly assess the impact of bacterial infection in causing KSHV reactivation through this mechanism [62].

Dai et al. reported that both bacterial PAMPs from *S. aureus* and *P. gingivalis* influenced KSHV entry into cells and subsequent expression of viral genes in primary human gingival fibroblasts (HDF) and periodontal ligament fibroblasts (PDLF) [63]. Dai et al. further explored if ROS was related to LTA/LPS facilitation of KSHV replication in oral cells [63,64]. They indeed found that LTA/LPS treatment significantly increased intracellular ROS production and NADPH oxidase activity in HGF and PDLF [63]. These results imply that bacteria might be the inducer of ROS production, and regulate KSHV lytic replication. Furthermore, LTA from *S. aureus* induced MAPK-ERK phosphorylation and LPS from *P. gingivalis* increased NF-κB phosphorylation in HGF [63]. It has previously been reported that once KSHV enters the cell, both the NF-κB and MAPK signaling pathways are required for successful establishment of latent infection [65,66]. However, in PDLF cells, both LTA- and LPS-induced NF-κB P65 had little effect on the MAPK pathways [63]. It is therefore apparent that the mechanism of PAMP-induced viral entry requires further investigation. The study did not look at the effects of TLR activation, which may be relevant as LTA and LPS are prominent ligands for TLR2 and TLR4, respectively. However, these results show that the microbiome in the oral cavity provide an amenable setting for KSHV infection and promote virus dissemination.

Dai et al. also examined the effects of *S. aureus* on KSHV reactivation [67]. *S. aureus* is a common, gram-negative intracellular pathogen that can colonize in the oral cavity of patients with periodontitis [68]. In both human gingival and periodontal ligament fibroblasts treated with the *S. aureus* medium, there were increased expression of KSHV lytic genes including RTA, vGPCR (ORF74), ORF-K8.1, and ORF57 [67]. Treatment with the *S. aureus* medium also induced release of infectious virion particles from both the gingival fibroblasts and PDLF. However, no significant effects from the medium of other gram-positive bacteria tested, such as *Bacillus subtilis* were observed, suggesting specific effect of the bacterial species. HIV+/KSHV+ patients also had higher levels of salivary LTA, a bacterial ligand for TLR2. However, the LTA ELISA kit used to analyze LTA levels did not distinguish LTA levels among different bacterial species. It would therefore be of interest to examine the effects of LTA from different gram-positive species on KSHV reactivation [67].

Mechanistically, Dai et al. found that *S. aureus* medium reduced the expression of KSHV microRNAs (miRNAs) compared with cells treated with control and fresh medium [67]. KSHV miRNAs play an important role in maintaining viral latency [67]. Furthermore, *S. aureus* medium downregulated expression of cyclin D1 and other host proteins responsible for processing cellular miRNAs, including Dicer, and Argonaut 1 and 2. These results suggested that the *S. aureus* medium might induce KSHV reactivation through the cyclin D1-Dicer-viral miRNAs axis in KSHV-infected oral cell lines [67]. The study also analyzed the effect of *S. aureus* and KSHV coinfection since *S. aureus* is an intracellular pathogen that can invade and survive in many cells including fibroblasts and endothelial cells. The authors concluded that coinfection in the same single cells induced KSHV reactivation. Moreover, a bacterial analysis of saliva samples in HIV+ patients showed 72% *S. aureus* compared to 37% in HIV-subjects [67]. This study is clinically relevant since methicillin-resistant *S. aureus* (MRSA) infection is a common complication in HIV patients [69]. Due to the ubiquitous nature of *S. aureus*, it may be worthwhile to investigate the effects of *S. aureus* in additional sites other than the saliva in HIV/KSHV-coinfected patients. Moreover, this study examined the conditioned medium of *S. aureus* which might contain many bacterial products besides LTA. It may be important to study the effects of purified LTA on KSHV-infected cells.

In a separate study, Dai et al. analyzed saliva samples for the presence of *P. gingivalis* and KSHV infection in HIV+ patients, and found that 11.3% were positive for both *P. gingivalis* (a periodontitis-associated bacterium) and KSHV [54,70]. There were also increased salivary LPS levels in KSHV-coinfected patients compared to patients without KSHV infection [54]. Mechanistically, Dai et al. found the *P. gingivalis* medium and LPS increased the expression of KSHV lytic genes in latently infected primary human oral fibroblasts through TLR4 [54]. However, authors suggested that additional bacterial products in the medium besides LPS might induce viral lytic gene expression, as blocking TLR4 expression did not completely abrogate the effect of *P. gingivalis* [54]. Likewise, *P. gingivalis* medium and LPS activated the MAPK pathways in KSHV-infected oral cells by inducing phosphorylation of p38 and JNK. However, treatment with p38 or JNK inhibitors did not completely hinder viral lytic reactivation, implying other pathways may be involved [54]. Interestingly, no significant effects were observed using medium or LPS from *E. coli* [54]. The differences in species-specific effects of LPS on KSHV reactivation is worth looking into. It is possible that the differences may be due to different LPS structures between the bacteria. The structure of the O-antigen as well as acylation patterns of the LPS structure may cause differences in receptor activation [71].

## 5. Impact of Bacteria on KSHV-Induced Cell Proliferation and Cellular Transformation

While lytic activation of KSHV is necessary for disseminating the infection, latency is essential for establishing persistent KSHV infection [40]. Upon KSHV infection, most cells remain in the latent state [72]. During latency, the virus expresses minimal proteins to evade the host immune response [40]. Epigenetic modifications of the viral genome and its associated histones play a crucial role in silencing viral lytic genes during latency [73]. Despite silenced expression of viral lytic proteins during latency, latent proteins are expressed resulting in cell proliferation by activating cell signaling pathways such as the STAT3 pathway, which is constitutively active in KSHV-infected cells [74,75]. Furthermore, during latency, KSHV utilizes the complement pathway to increase cell survival [76]. As the complement system has a central role in the defense against pathogens, it is particularly relevant to assess the bacterial effects on KSHV latently-infected cells in addition to lytic reactivation [9].

Gruffaz et al. analyzed the expression of TLRs in rat primary embryonic metanephric mesenchymal precursor (MM) cells and KSHV-transformed MM (KMM) cells [77]. Multiple TLRs were upregulated in KMM cells compared to MM cells [77]. Notably, TLR4 was upregulated by over 40 times in KMM cells compared to MM cells [77]. Authors also observed that TLR4 was upregulated in KSHV-infected spindle tumor cells in human KS lesions and KSHV-infected TIME cells [77]. Mechanistically, Gruffaz et al. reported that KSHV-upregulated expression of TLR4 was mediated by multiple viral miRNAs, and that activation of the TLR4 pathway in KSHV-infected cells results in chronic induction of cytokines IL6, IL1β, and IL18 [77]. The study also demonstrated that IL6 mediated constitutive activation of the STAT3 pathway [77]. These results emphasized the essentiality of TLR4 pathway in KSHV-induced cellular transformation and tumorigenesis, underscoring the impact of bacterial infection in exacerbating KSHV-induced pathology.

Markazi et al. further investigated the effects of TLR4 by stimulating MM/KMM and BJAB/KSHV-infected BJAB cells (EBV-negative B-cell lymphoma cells) with *Pseudomonas aeruginosa* [78]. *P. aeruginosa* is a gram-negative opportunistic pathogen that can infect immunosuppressed individuals [79]. *P. aeruginosa* infection resulted in increased proliferation in KSHV-infected BJAB and KMM cells but had no significant effects in the KSHV uninfected control cells [78]. As KMM cells have previously been shown to form colonies in soft agar [80], Markazi et al. demonstrated that *P. aeruginosa* stimulation increased cell proliferation and efficiency of colony formation in soft agar of KMM cells but had no significant effect on the untransformed MM cells [78]. Mechanistically, the study showed that *P. aeruginosa* increased inflammatory cytokines and activation of p38, ERK1/2, and JNK MAPK pathways in KMM cells through LPS and flagellin ligands [78]. These results imply that opportunistic infection is likely an important target in mitigating KS pathology. Furthermore, this study shows that it is necessary to assess individual bacterial PAMPs on the activation of cell signaling pathways in order to more effectively elucidate molecules involved in inflammation and identify the potential therapeutic targets for KSHV-induced cancers. In this study, LPS and flagellin ligands were assessed for their effects on inducing inflammation in KSHV-infected cells. However, bacteria possess many other PAMPs (lipoproteins, LTA, peptidoglycans, etc.) and secrete by-products (H_2_O_2_, SCFAs, quorum signaling molecules, etc.) that may induce proliferation in KSHV-latently infected cells, and therefore may be worth further investigation.

## 6. Conclusions and Future Perspectives

The collection of studies reviewed herein has demonstrated that bacteria can affect KSHV-induced cancers by numerous mechanisms, including through bacterial PAMPS and bacterial-secreted byproducts. Indeed, microbiome analyses reveal increased levels of pathogenic bacteria and secreted PAMPs in immunosuppressed HIV/KSHV-coinfected patients [51,54,67]. Bacteria can reactivate KSHV through both direct and indirect methods. Bacterial species such as *F. nucleatum* and *P. gingivalis* produce SCFAs which suppress HDAC activity, resulting in increased hyperacetylation and subsequent expression of lytic gene expression [53,55,56]. Bacteria can also induce inflammation through PAMPs which may reactivate KSHV indirectly through increased ROS production [63,64,67]. KSHV reactivation leads to the spread and dissemination of the virus [40]. Moreover, recent studies have demonstrated that inflammation induced by bacterial LPS and flagellin PAMPs may enhance cell proliferation and KSHV-induced cellular transformation in the latently infected cells [77,78]. Analyzing the effects of bacteria on latently infected cells is especially relevant since the majority of KSHV-infected cells within KS tumors are in the latent state [72]. By elucidating the various mechanisms of bacteria-induced effects on KSHV-infected cells, researchers gain important insights into the cell signaling pathways involved in KSHV replication and dissemination as well as increased survival and proliferation of tumor cells.

Since interactions among bacterial species and the host are complex, it is crucial to more thoroughly investigate the microbial signature of HIV/KSHV-coinfected patients to enhance our understanding of bacterial biomarkers and the underlying mechanisms that contribute to KSHV pathology. Many studies have reported that immunosuppression and inflammation drive KSHV pathology, both of which are applicable to bacterial infection [4]. Interestingly, there is a paucity of data assessing the effects of KSHV infection on microbiome dysregulation. However, since KSHV encodes multiple proteins (e.g., K3, K5, LANA, and RTA) that interfere with the host immune response, it can be speculated that KSHV might alter the host microbiome by deregulating immune response [81].

While the majority of microbiome analyses in HIV/KSHV-coinfected patients used saliva samples, it may be beneficial to assess the microbiome in additional sites, such as KS lesions present on different organs (skin, lungs, intestines, lymph nodes, etc.) as microbial species vary depending where they are located [31]. Moreover, while studies have reported higher levels of bacterial PAMPs in immunosuppressed patients, including LPS, LTA, and flagellin, the kits used to analyze these PAMPs do not distinguish between different bacterial species [19,67]. There is evidence that the same types of PAMPs (i.e., LPS) from different bacterial species can have varying effects on the immune responses [71]. Hence, it might be important to delineate the distinct effects of specific bacterial species on KSHV-induced cancers.

It has been demonstrated that the NF-κB, STAT3, and MAPK pathways play crucial roles in KSHV reactivation and cell proliferation of KSHV latently infected cells [55,63,77,78]. It can be speculated that activation of MAPK pathways by bacteria can increase KSHV infectivity and viral dissemination [57,59,60] while bacteria-induced inflammation can also promote tumorigenesis through the STAT3 pathway [77]. However, as there are numerous PRRs and downstream cascades associated with bacterial infection, it is highly likely that additional pathways could be involved in promoting KS pathology. The effects of bacteria on the immune response are highly intricate, and pathways including cytoplasmic bacterial immune sensors such as nod-like receptors or the cGAS-STING pathway have not yet been explored in relation to bacterial effects on KSHV infection and KSHV-induced cell proliferation [6,82]. Furthermore, although the studies presented in this review examine the activation of pro-survival pathways by bacterial PAMPs and metabolic products, there are additional bacterial products that may activate similar pathways in KSHV-infected cells. For example, several studies report that bacterial biofilms and quorum signaling molecules can induce inflammatory immune responses; hence it may be worth investigating their effects on promoting KSHV-induced cancers [83,84,85]. Thorough understanding of bacterial PAMPs and associated cell signaling pathways involved in KSHV pathogenesis is essential for discovering novel molecular targets for therapeutic intervention of KSHV-induced cancers.

The utilization of KSHV-infected cells in cell culture experiments has been proven invaluable for providing data on the effects of bacteria on KS pathology. The KSHV-infected cells used in both the Gruffaz et al. and Markazi et al. studies were developed by infecting primary rat embryonic metanephric mesenchymal precursor cells [77,78,80]. This resulted in complete cellular transformation characterized by immortalization, colony formation in soft agar, and tumor induction in mice [80]. Although in vitro experiments have revealed that bacteria can regulate cell proliferation and viral replication of KSHV-infected cells, the effect of bacteria on promoting KSHV infection and the process of cellular transformation have not yet been examined. Since KSHV-associated bacteria can activate mitogenic pathways necessary for KSHV primary infection, it is plausible that bacteria might promote cellular transformation as well [57,77,78].

The collection of studies explored in this review focus predominantly on pathogenic bacteria enhancing KSHV-induced cancer pathology. However, bacteria explored as anti-cancer therapies have also been of interest for multiple cancer types [25,26]. Moreover, abundant evidence demonstrates that certain bacterial species (e.g., probiotics) can mitigate cancer pathology [3]. Probiotics may enhance immunity against cancer through multiple mechanisms including out competition of pathogenic bacteria, degradation of carcinogens, and production of anti-inflammatory mediators [3]. Although Gruffaz et al. reported increased levels of the *Firmicutes* phylum in KS patient, *Firmicutes* bacteria are potent producers of SCFA such as butyrate, which can reactivate KSHV and other herpesviruses [51,55,86]. This feature of *Firmicutes* bacteria potentially complicates the use of probiotic bacteria such as *Lactobacillus* as “anti-inflammatory” bacteria as they can be potent producers of SCFAs [3]. Further inquiry into the effects of therapeutic bacteria on KSHV-driven diseases may be critical in future studies.

Notwithstanding the potential of anti-tumor bacterial species, the studies explored in this review strongly emphasize the negative effects of pathogenic, opportunistic infection in patients with KSHV-induced cancers. As these cancers continue to be the most common cancer types in immunosuppressed patients despite increased use of antiretroviral drugs, it is evident that more efficient treatments are in demand [37]. Microbiome research in relation to KSHV-induced cancers is undoubtedly a crucial step in pioneering enhanced therapies for mitigating KSHV pathology.
